# Neutrophils Infiltrate the Spinal Cord Parenchyma of Rats with Experimental Diabetic Neuropathy

**DOI:** 10.1155/2017/4729284

**Published:** 2017-02-15

**Authors:** Victoria L. Newton, Jonathan D. Guck, Mary A. Cotter, Norman E. Cameron, Natalie J. Gardiner

**Affiliations:** ^1^Division of Diabetes, Endocrinology and Gastroenterology, School of Medical Sciences, Faculty of Biology, Medicine and Health, University of Manchester, Oxford Road, Manchester M13 9PT, UK; ^2^School of Medical Sciences, University of Aberdeen, Aberdeen, UK

## Abstract

Spinal glial cell activation and cytokine secretion have been implicated in the etiology of neuropathic pain in a number of experimental models, including diabetic neuropathy. In this study, streptozotocin- (STZ-) induced diabetic rats were either untreated or treated with gabapentin (50 mg/kg/day by gavage for 2 weeks, from 6 weeks after STZ). At 8 weeks after STZ, hypersensitivity was confirmed in the untreated diabetic rats as a reduced response threshold to touch, whilst mechanical thresholds in gabapentin-treated diabetic rats were no different from controls. Diabetes-associated thermal hypersensitivity was also ameliorated by gabapentin. We performed a cytokine profiling array in lumbar spinal cord samples from control and diabetic rats. This revealed an increase in L-selectin, an adhesion molecule important for neutrophil transmigration, in the spinal cord of diabetic rats but not diabetic rats treated with gabapentin. Furthermore, we found an increase in the number of neutrophils present in the parenchyma of the spinal cord, which was again ameliorated in gabapentin-treated diabetic rats. Therefore, we suggest that dysregulated spinal L-selectin and neutrophil infiltration into the spinal cord could contribute to the pathogenesis of painful diabetic neuropathy.

## 1. Introduction

The prevalence of diabetes mellitus is increasing worldwide, which has significant health and economic implications. Associated costs include both primary treatment and also treating the associated secondary complications such as retinopathy, nephropathy, and neuropathy [1]. Distal sensory polyneuropathy (DPN) is the most common of the peripheral nerve disorders associated with diabetes [2]. DPN may be accompanied by paresthesia such as tingling and burning sensations, heightened sensitivity to normally innocuous stimuli, and spontaneous pain. Neuropathic pain serves no useful function [3] and is a debilitating condition [4]. There is currently no effective treatment, with single analgesics usually failing to adequately treat the pain, meaning that patients are offered available therapies in a stepwise and often combinatorial fashion [5, 6]. One of the first-line treatments is gabapentin [7], an analogue of the inhibitory neurotransmitter *γ*-aminobutyric acid (GABA), an anticonvulsant drug which is also antiallodynic in neuropathic and inflammatory pain states [8]. The underlying mechanisms of analgesia provided by this drug are not thoroughly understood. However, gabapentin can interact with several targets, including the *α*2-*δ* subunit of voltage-activated calcium channels [9] and the L-amino acid transporters (LAT-1 and LAT-2; [10]) and has been shown to reduce spinal microglial activation and allodynia in rats with experimental diabetes [11, 12].

The pathogenesis of neuropathic pain involves changes in neuronal activity in the peripheral and central nervous systems and also activation of glial and immune cells [13, 14]. Glial cells are rapidly activated in response to peripheral nerve injury [15–17] and are responsible for the release of many inflammatory mediators, including chemokines and cytokines important for the establishment and maintenance of neuropathic pain [13, 18]. In rodent models of diabetes, microglia are activated in the dorsal horn of the lumbar spinal cord [11, 19, 20] and their activation has been associated with neuropathic pain. Whilst the intact blood-brain barrier largely prevents circulating immune cells from crossing into the parenchyma, permeability can be modulated by trauma or inflammation, enabling recruitment and infiltration of immune cells into the central nervous system (CNS). Peripheral injection of carrageenan into the hind paw, for example, causes an increase in ICAM and VCAM expression in spinal epithelium, changes in tight junction proteins, an increase in brain-barrier permeability, and the consequent migration of neutrophils into the CNS [21]. In both diabetic nephropathy and retinopathy, increased leukostasis and infiltration have been observed and are detrimental to symptom severity [22, 23]. However, to our knowledge, no information exists regarding the transmigration of neutrophils in the spinal cord in painful diabetic neuropathy.

In this study, we identify an increase in L-selectin and in the number of neutrophils present in the parenchyma of the spinal cord of untreated diabetic rats, which could represent a novel therapeutic target to explore in future studies.

## 2. Methods

### 2.1. Induction of Diabetes and Drug Delivery

All experiments were carried out using mature adult male Sprague-Dawley rats (495–570 g, bred at The Institute of Medical Science, Aberdeen University) in accordance with the UK Animals (Scientific Procedures) Act 1986. Diabetes was induced using streptozotocin (STZ in sterile saline; 40 mg/kg i.p.; *n* = 12). Age- and weight-matched rats were used as nondiabetic controls (*n* = 6). Diabetic rats were either untreated or treated daily for 2 weeks with gabapentin (50 mg/kg/day by gavage; Spectrum Chemical Manufacturing Corp, Gardena) starting 6 weeks following STZ injection.

### 2.2. Behavioural Testing

After 8 weeks of diabetes and within 2 hours following gabapentin administration, the behavioural response to mechanical and thermal stimulation of the hind paw was assessed using an automatic Von Frey probe and Hargreaves apparatus, respectively (Ugo Basile, Italy). All animals were habituated to the tester and the environment prior to testing. The Von Frey probe was set to deliver an initial 2 g force over 8 seconds. The stimulus was then increased by a 0.2 log unit increment if there was no response or decreased by the same increment if the animal responded. Testing was performed on each foot and a 50% threshold was determined [24, 25]. An average value for the 2 feet was then calculated. For thermal testing, the time taken for the animal to remove its hind paw from the infrared heat stimulus was recorded 3 times for each foot. The first reading from each foot was discarded, and the mean response time was then calculated.

### 2.3. Tissue Processing

Rats were killed by isoflurane overdose 1 day after their final behavioural assessment and dose of gabapentin. Blood glucose levels were measured using an Ascensia Breeze 2 machine (Bayer, UK) from blood obtained by cardiac puncture. The lumbar (L3–L6) spinal cord region was rapidly dissected and postfixed in ice-cold 4% paraformaldehyde for 4 hours or frozen on dry-ice.

### 2.4. Immunohistochemistry

Fixed lumbar spinal cord was cryoprotected at 2–8°C in 10% sucrose in 0.1 M phosphate buffer for 18–24 hours followed by 30% sucrose in 0.1 M phosphate buffer for a further 18–24 hours. Tissue was then embedded in OCT embedding matrix media (Thermo Shandon Ltd., UK) and frozen on dry-ice. Transverse sections were cut by cryostat (16 *μ*m) and thaw-mounted onto Superfrost Plus Slides (Fisher Scientific, UK).

For staining of microglia, one group of slides were first incubated twice for 15 minutes each (0.1 M PBS, 20% (v/v) methanol, 1.5% (v/v) hydrogen peroxide) for peroxidase-conjugated studies. After washing (phosphate buffered saline (PBS)), nonspecific binding in all slides was blocked for an hour (5% goat serum in 0.2% triton-X in PBS at room temperature) and sections were incubated overnight with primary antibody for ionized calcium binding adaptor molecule 1 (rabbit anti-Iba1; Wako, Germany; 1/1000 in 5% goat serum in 0.2% triton-X PBS at 2–8°C;). Biotinylated or fluorescein conjugated anti-rabbit secondary antibody (1/500 in 5% goat serum in 0.2% triton-X PBS; Vector, USA) was allowed to bind primary antibody for 1.5 hours at room temperature. Fluorescent sections were washed then either mounted in Vectorshield containing DAPI (Vector, USA) or incubated for 1 hour at room temperature with AvidinBiotin solution as per manufacturer's instructions (VECTASTAIN® ABC system, Vector, USA). Immunoreactivity was then visualised using DAB as per manufacturer's instructions (Vector, USA). Sections were dehydrated through a 50, 70, 90%, and absolute ethanol series followed by xylene washes, before mounting with Pertex mounting media (CellPath, UK).

For staining of neutrophils and blood vessels, sections were washed and nonspecific binding and autofluorescence were reduced (0.1 M glycine, 5% goat serum, 5% donkey serum in 0.3% triton-X in PBS; for 2 hours at room temperature). Primary antibodies for rabbit anti-neutrophil serum (SJC; 1/10,000; gift A. Dénes, (University of Manchester, UK) from D. Anthony and S. Campbell, University of Oxford, UK, produced [26]) and mouse anti-human laminin (1/1000; Millipore, UK) were applied overnight (in 2.5% goat serum, 2.5% donkey serum 0.3% triton-X in PBS, 2–8°C). After washing, primary antibodies were visualised with donkey anti-rabbit secondary Cyanine- 3-(Cy3-) conjugated antibody (1/1000; Jackson ImmunoResearch, USA) and goat anti-mouse conjugated Alexa Fluor™ 488 (1/1000; Invitrogen Life Technologies, UK) for 2 hours at room temperature (5% goat serum, 5% donkey serum in 0.3% triton-X in PBS).

Negative control sections were produced with each batch of immunostaining, with the absence of primary antibody. Staining was visualised on a Leica DMR microscope and images captured using a Hamamatsu digital C4742-95 digital camera (Japan). Higher power fluorescence images were taken using an Olympus BX51 upright microscope and Coolsnap EZ (Photometrics, USA) camera running MetaVue Software (Molecular Devices, USA), or a Delta Vision (Applied Precision, USA) restoration microscope using a 60x objective, captured using a Coolsnap HQ (Photometrics, USA) camera with a *Z* optical spacing of 0.2 *μ*m. Raw images were then deconvolved using Softworx (Applied Precision, USA). High magnification images of microglia were captured on a Panoramic 250 slide scanner (3DHISTECH Ltd., Hungary), using a 20x objective and visualised using Pannoramic Viewer 1.15 software (3DHISTECH Ltd., Hungary).

### 2.5. Cytokine Profile Array

Spinal cords were homogenized in ice-cold lysis buffer (25 mM Tris HCl pH 7.4, 15 mM NaCl, 10 mM NaF, 10 mM Na Pyrophosphate, 2 mM EDTA, 0.2 mM Na_4_OV_3_, 1 mM PMSF, Protease Inhibitor Cocktail (1 : 200; Sigma Aldrich, UK)) using the FastPrep® bead beater system (MP Biomedicals, USA). The protein concentration of each sample was determined using a BCA assay (Pierce Biotechnology, UK) and 350 *μ*g of lysate was loaded per membrane (Rat Cytokine Array Panel A, R&D Systems, UK), as per manufacturer's instructions. Cytokine levels were visualised and quantified using IRDye® 800CW Streptavidin secondary antibody (1/2000 in assay buffer 6; Li-Cor, UK) and captured on an Odyssey® Infrared Imaging System. Results were analysed using Odyssey 2.1 Software (Li-Cor, UK) and all absorbance values were corrected for background.

### 2.6. L-Selectin ELISA

Spinal cord lysates from control, untreated diabetic and gabapentin-treated rats (75 *μ*g of protein) were loaded in duplicate and analysed for L-selectin content using a commercial ELISA kit (rat L-selectin/CD62L DuoSet, R&D Systems, UK).

### 2.7. Spinal Cord Neutrophil Analysis

To determine the number of infiltrating neutrophils, dual labelling was used to visualise both blood vessels and neutrophils present within the parenchyma. Only neutrophils outside of blood vessels were measured, that is, not intraluminal neutrophils. Parenchymal cell counts were performed on 4-5 lumbar spinal cord sections per animal (*n* = 6) and expressed as mean number of neutrophils per section.

### 2.8. Microglia Analysis

Iba1-immunoreactive cell morphology was analysed from DAB-labelled sections using the method described by Calvo et al. [27]. Briefly, SigmaScan Pro software (SPSS) was used to overlay a grid with squares measuring 10000 *μ*m^2^ on regions of the dorsal horn. A minimum of 5 randomly selected squares from a minimum of 4 spinal cord sections per animal were analysed. Only cells with a clearly visible cell body were analysed. Microglia whose processes were greater than double the cell-body length were deemed “surveying.” Those cells where processes were less than double the cell-body length were categorised as “effector.” Results are presented as the percentage of microglia displaying an “effector” morphology. Iba-1-immunoreactive cells present in the superficial dorsal horn were counted from 3–5 sections per animal and expressed as number of cells per 1 × 10^5^ *μ*m^2^.

### 2.9. Statistical Analysis

Data are expressed as mean values ± standard deviation, unless otherwise stated. A Kruskal-Wallis ranking test was carried out on the Von Frey, and L-selectin ELISA data and 1-way ANOVA with Bonferroni post hoc tests were used to analyse all other data using GraphPad Prism 4 software (GraphPad software, USA).

## 3. Results

### 3.1. Gabapentin Reduces Hypersensitivity in STZ-Diabetic Rats

**Figure 1:**
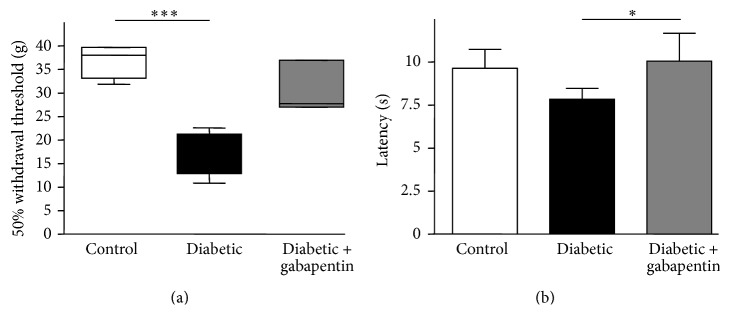
Gabapentin corrects diabetes-induced hypersensitivity. (a) Mechanical allodynia was observed in untreated diabetic rats (8 weeks after STZ), whilst diabetic rats treated with gabapentin showed near-control thresholds. Data are displayed as box and whisker plots, ^*∗∗∗*^*p* < 0.001, in a Kruskal-Wallis test with Dunn's post hoc tests. (b) Thermal hyperalgesia was determined using a Hargreaves device; again diabetic rats treated with gabapentin showed near-control thresholds ^*∗*^*p* < 0.05, in a 1-way ANOVA with Bonferroni post hoc tests. Data represent mean latency + SD; *n* = 6.

Rats with STZ-induced diabetes displayed mechanical allodynia at 8 weeks compared with age-matched controls (control: 37.0 g ± 3.2 versus untreated diabetic: 17.4 g ± 4.1, *p* < 0.001; Figure 1(a)). Gabapentin-treated diabetic rats (50 mg/kg/day for 2 weeks from 6 weeks after STZ) did not demonstrate the same degree of mechanical sensitivity, as no significant difference was observed between control and gabapentin-treated diabetic rats at 8 weeks (30.6 g ± 5.0, Figure 1(a)). In addition, diabetes-associated thermal hyperalgesia was prevented with gabapentin (untreated diabetic: 7.8 s ± 0.6 versus gabapentin-treated diabetic: 10.1 s ± 1.6, *p* < 0.05; Figure 1(b)). Gabapentin therefore protects against diabetes-associated allodynia and thermal hyperalgesia, without affecting terminal blood glucose levels (control: 8.1 ± 0.4 mmol/L; untreated diabetic: 34.1 ± 4.8 mmol/L (*p* < 0.001); diabetic + gabapentin: 39.2 ± 7.1 mmol/L (*p* < 0.001) compared with blood glucose levels in control rats: 1-way ANOVA with Bonferroni post hoc tests).

### 3.2. Diabetes-Induced Increase in L-Selectin in the Spinal Cord is Prevented by Gabapentin

**Figure 2:**
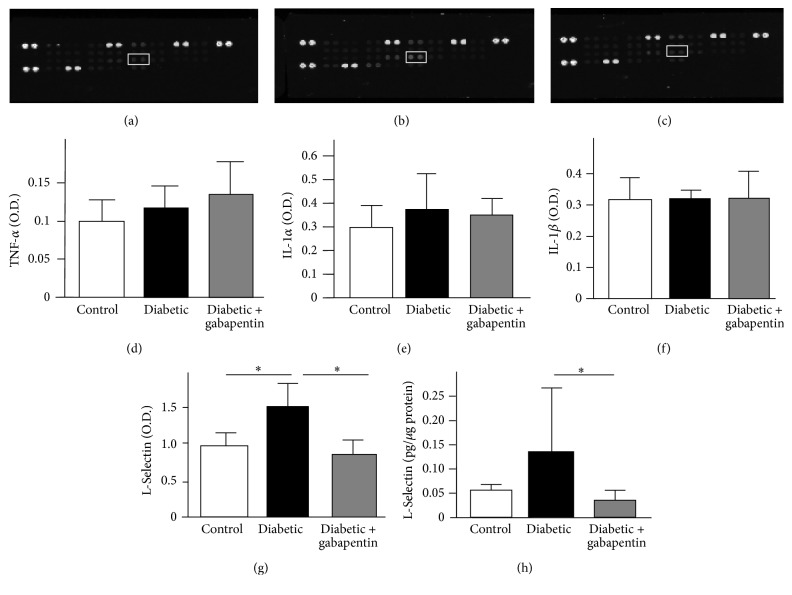
L-Selectin is increased in the spinal cord of diabetic rats. Cytokine proteome arrays were used to assay levels of 29 cytokines in (a) control, (b) untreated diabetic, and (c) gabapentin-treated diabetic rats. There was no change in the spinal levels of classically proinflammatory cytokines such as (d) TNF-*α*, (e) IL-1*α*, or (f) IL-1*β* (g). However, L-selectin (highlighted in box in (a)–(c)) was significantly increased in diabetic rat lumbar spinal cord, and this increase was ameliorated by treatment with gabapentin (^*∗*^*p* < 0.05 in a 1-way ANOVA with Bonferroni post hoc tests. Mean optical densities + SD are displayed; *n* = 4). (h) This was independently confirmed with an ELISA; levels of L-selectin were reduced when diabetic rats were treated with gabapentin (^*∗*^*p* < 0.05 in a Kruskal-Wallis test with Dunn's multiple comparison test; *n* = 5-6).

An increase in the levels of proinflammatory cytokines has previously been reported in the rat spinal cord at early time points of diabetes (4-5 weeks after STZ: [28, 29]). We therefore conducted a cytokine profiling array using lumbar spinal cord samples (Figures 2(a)–2(c); Table 1) to determine which were altered at the 8-week time point of diabetes. The levels of classical “proinflammatory” cytokines such as TNF-*α* (Figure 2(d)), IL-1*α* (Figure 2(e)), and IL-1*β* (Figure 2(f)) were not significantly different in spinal cord samples from diabetic rats. Indeed, the only significant change (the full panel of results of 29 cytokines is shown in Table 1) was in the expression of L-selectin, which was significantly increased by 55% in spinal cords from untreated diabetic rats compared to control rats; this increase was prevented by gabapentin (Figure 2(g)). This observation was validated using ELISA (untreated diabetic: 0.14 ± 0.13 *ρ*g L-selectin/*μ*g spinal cord protein versus gabapentin-treated diabetic: 0.04 *ρ*g ± 0.02 L-selectin/*μ*g spinal cord protein; *p* < 0.05; Figure 2(h)).

### 3.3. Glial Cell Profiling in the Dorsal Horn of Rats with 8 Weeks of Experimental Diabetes

**Figure 3:**
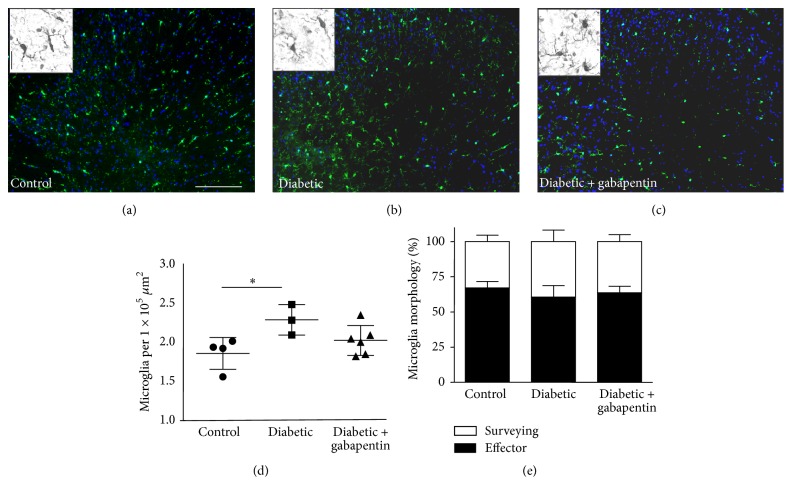
Microglial changes in the spinal cord of control, untreated diabetic, and gabapentin-treated diabetic rats. Representative photomicrographs of the L4/5 dorsal horn of the spinal cord of (a) control, (b) untreated diabetic, and (c) diabetic rats treated with gabapentin (a–c), micrographs (and insets) show examples of typical Iba1-immunoreactivity. Scale bars represent 100 *μ*m (main micrograph, (a)–(c)) and 25 *μ*m (insets (a)–(c)). Microglial numbers are increased in the superficial ((d),^*∗*^*p* < 0.05 1-way ANOVA Bonferroni post hoc tests) but not deep (e) dorsal horn of diabetic rats; however, morphology of microglia was not significantly different (e).

A change in spinal microglial number and morphology has previously been reported at early time points of diabetes (2–4 weeks after STZ: [11, 20]). Here, we examined Iba-1-immunopositive microglia at a later time point in the lumbar spinal cord of control, untreated diabetic, and diabetic rats treated with gabapentin (Figure 3). At 8 weeks after STZ, there was a small but significant increase in the numbers of Iba-1-positive cells present in the superficial dorsal horn of untreated diabetic rats (Figures 3(a)–3(d); *p* < 0.05 1-way ANOVA with Bonferroni post hoc tests); however, cell morphology was equivalent in all treatment groups (Figure 3(e); control: 67%  ± 4.6: untreated diabetic: 61%  ± 8.1; gabapentin-treated diabetic 63%  ± 4.8 microglia had “effector” morphology; i.e., processes were less than twice the length of the cell body).

### 3.4. Increased Numbers of Neutrophils Are Present in the Spinal Cord Parenchyma of Diabetic Rats, Which Is Ameliorated with Gabapentin Treatment

**Figure 4:**
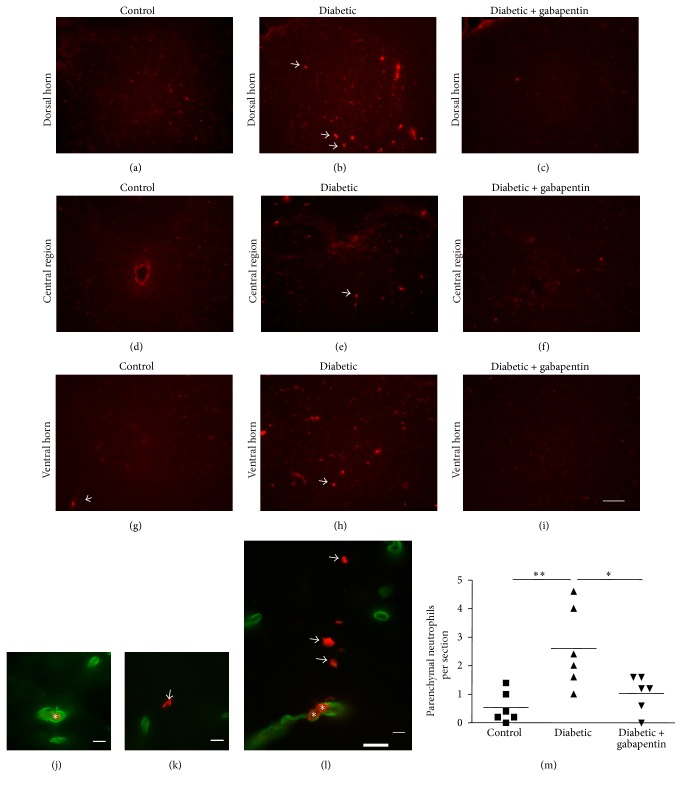
Diabetes is associated with an increase in neutrophil numbers within the spinal cord parenchyma, which is ameliorated with gabapentin treatment. (a–i) There is an increase in antineutrophil immunoreactivity within the dorsal horn (a–c), central region (d–f), and ventral horns (g–i) of untreated diabetic rats (arrows, (b), (e), and (h), scale bar represents 100 *μ*m.). Dual immunofluorescence with antilaminin (green) to label blood vessels reveals the presence of intraluminal (asterisks, (j) and (l)) and parenchymal neutrophils (arrows, (k) and (l)). Only parenchymal neutrophils (i.e., those not associated with a vessel) were counted (m) and revealed an increase in neutrophils in the spinal cord of untreated diabetic rats and revealed that gabapentin treatment reduced the numbers to near-control levels ((m), ^*∗*^*p* < 0.05, ^*∗∗*^*p* < 0.01 in a 1-way ANOVA with Bonferroni post hoc tests. *n* = 6).

The diabetes-associated upregulation of L-selectin, an adhesion molecule important for neutrophil transmigration [30], suggested possible immune cell recruitment/infiltration into the spinal cord. Therefore, immunohistochemistry was performed to identify whether neutrophils had infiltrated the spinal cord. The spinal cord parenchyma of untreated diabetic rats (arrows, Figures 4(b), 4(e), and 4(h)) contained more neutrophils than control (Figures 4(a), 4(d), and 4(g)) and gabapentin-treated (Figures 4(c), 4(f), and 4(i)) rats. Since rats were not transcardially perfused prior to tissue harvest, only neutrophils present in spinal parenchyma (arrows, Figures 4(k) and 4(l)) were counted and not those contained within blood vessels (asterisks, Figures 4(j) and 4(l)). There was a significant increase in the number of parenchymal neutrophils throughout the spinal cord in untreated diabetic rats compared with control rats (untreated diabetic: 2.60 ± 1.4 versus control: 0.53 ± 0.55; Figure 4(m)  *p* < 0.01). Interestingly, this increase was not observed in diabetic animals treated with gabapentin (1.03 ± 0.63, *p* < 0.05 compared with untreated diabetic rats).

## 4. Discussion

In this study, we demonstrate an increase in the levels of L-selectin, an adhesion molecule important for neutrophil transmigration, in the lumbar spinal cord after 8 weeks of diabetes. In addition, we show an increase in the number of parenchymal neutrophils in the spinal cord of diabetic rats. Together, these data suggest a role for dysregulated L-selectin and spinal vasculature in diabetes that leads to an increase in infiltrating neutrophils in experimental diabetic neuropathy, which is ameliorated by treatment with gabapentin.

The cytokine profiling array did not show diabetes-associated increases in classic proinflammatory cytokines such as IL-1*β*, TNF-*α*, fractalkine, and IL-6 cytokines, which are associated with microgliosis and hyperalgesia [11, 20, 28, 29, 31, 32]. Microglial activation has been associated with pain in a number of neuropathy models, notably traumatic nerve-injury models [14] and early time points of STZ-diabetes [11, 31]. However, microgliosis was not associated with viral-induced hypersensitivity [33] and minimal in a nucleoside reverse transcriptase inhibitor- (stavudine-) induced HIV hypersensitivity model [34]. Our finding that there was a small increase in microglial number, but no change in morphology at 8 weeks, perhaps indicates why we did not detect increases in the proinflammatory cytokines in our arrays but does not preclude the possibility that microglial activation may have occurred at an earlier time point. It is also possible that subtle changes in proinflammatory cytokines specifically within the dorsal horn may have not been detected, as whole lumbar spinal cord lysates were used in our assay.

However, interestingly, we found a significant increase in L-selectin (a type I cell adhesion molecule which is constitutively expressed on the cell surface of most circulating leukocytes [30]) in spinal cords from diabetic rats, which was not evident in spinal cords from diabetic rats treated with gabapentin. L-Selectin mediates the initial tethering and rolling of leukocytes along activated endothelial cells and then the transendothelial migration of leukocytes through the vasculature. L-Selectins are cleaved from the cell surface by the action of ADAM17 (disintegrins and metalloproteinase 17) or TACE (TNF-*α* converting enzyme), a process known as “shedding,” which may dramatically regulate migratory cell behaviour [35].

Neutrophils are one of the first types of peripheral immune cell to attend sites of inflammation following injury or infection [36]. They are important in mounting an initial immune response, able to produce both proinflammatory mediators, as well as present antigen to T-cells [37]. In experimental nerve-injury models, the recruitment of immune cells both peripherally and centrally is an important mechanism underlying establishment and maintenance of neuropathic pain [38]. Neutrophil migration into the CNS is well characterised in spinal cord injury models [39], and reductions in leukocyte infiltration in the CNS have been linked to reductions in neuropathic pain [40]. The reactive glycolytic metabolite methylglyoxal is increased in the plasma of both STZ-diabetic mice and diabetic patients [41]. Interestingly, peripheral injection of methylglyoxal induces leukocyte recruitment to the microvasculature of the injection site and approximately 90% of the recruited cells are neutrophils [42], suggesting that recruitment of this cell type may be particularly important in diabetes. In diabetic retinopathy, higher numbers of CD45^+^ immune cells have been recorded in the retina of diabetic mice compared with controls [43]. In diabetic rats too, increased numbers of leukocytes are associated with retinal endothelial cells, when compared with retina from control rats [44]. Similarly, an increase in neutrophils in the kidney has been described in mice with diabetic nephropathy [45]. Increased numbers of neutrophils in the parenchyma of spinal cords from diabetic rats compared with controls indicate commonality between the three major diabetic complications.

This increase in spinal neutrophil recruitment in diabetes could be a consequence of altered afferent activity, spinal sensitisation, structural damage, or altered blood-brain barrier function. Interestingly, the reduction in numbers of neutrophils within the spinal cord parenchyma after gabapentin followed the reduction in allodynia and thermal hyperalgesia in diabetic rats. The reduced numbers of spinal neutrophils in diabetic rats treated daily with gabapentin may be a downstream consequence of the transitory analgesic effects of gabapentin ([46]). This may be through inhibition of excitatory postsynaptic currents from dorsal horn neurons or a reduction in microgliosis persisting for a sufficient length of time to prevent neutrophil infiltration into the spinal cord. It will be interesting for future studies to examine whether gabapentin has a direct effect on L-selectin expression. This effect may be via the spinal vasculature, neutrophil migration across the endothelium, and/or release from the bone marrow. Indeed, in an induced paw edema model, gabapentin treatment reduced both leukocyte counts and MPO activity (neutrophil marker) in the foot [47]. Depletion of circulating neutrophils has also previously been shown to attenuate the development of hyperalgesia following a peripheral nerve injury [48]. We suggest that future studies will be important to determine the impact of blocking neutrophil recruitment to the spinal cord on neuropathic pain in diabetes. Using intrathecal L-selectin function-blocking antibodies or inhibitors may provide valuable mechanistic evidence linking spinal L-selectin expression and neutrophil invasion with the pathogenesis of diabetic neuropathy.

In conclusion, this current work highlights the importance of considering not only glial activation, changes in ion channel expression, and altered neuronal activity in painful diabetic neuropathy, but also the effect of immune cell infiltration into the spinal cord; this may open more avenues by which to direct future therapeutic targeting.

## Figures and Tables

**Table 1:** Rat spinal cord cytokine proteome array. The levels of 29 cytokines in the lumbar spinal cord of control, diabetic, and gabapentin-treated diabetic rats. Mean values ± SD are displayed. *n* = 4. ^*∗*^*p* < 0.05 in a 1-way ANOVA.

Name	Mean OD ± SD						*p* value
	Control		Diabetic		Diabetic + gabapentin		
IL-1*α*	**0.30**	0.09	**0.37**	0.15	**0.35**	0.07	*0.60*
IL-1*β*	**0.32**	0.07	**0.33**	0.03	**0.32**	0.09	*0.96*
IL-1ra	**0.30**	0.03	**0.37**	0.02	**0.33**	0.05	*0.09*
IL-2	**0.25**	0.03	**0.26**	0.06	**0.36**	0.09	*0.10*
CINC-1	**0.39**	0.21	**0.51**	0.34	**0.56**	0.24	*0.68*
CINC-2	**0.10**	0.02	**0.12**	0.02	**0.13**	0.05	*0.40*
CINC-3	**0.31**	0.04	**0.37**	0.04	**0.33**	0.02	*0.12*
IFN-*γ*	**0.31**	0.05	**0.29**	0.02	**0.27**	0.02	*0.19*
IL-3	**0.26**	0.09	**0.26**	0.05	**0.28**	0.08	*0.91*
IL-4	**0.23**	0.08	**0.25**	0.06	**0.27**	0.08	*0.74*
IL-6	**0.25**	0.04	**0.24**	0.03	**0.20**	0.04	*0.26*
IL-10	**0.25**	0.08	**0.22**	0.03	**0.24**	0.04	*0.82*
CNTF	**6.52**	1.91	**8.68**	2.50	**9.49**	3.47	*0.32*
sICAM-1	**9.18**	3.28	**13.10**	2.74	**12.80**	2.63	*0.15*
Thymus chemokine	**19.90**	0.36	**23.20**	3.03	**17.10**	5.85	*0.13*
Fractalkine	**1.02**	0.24	**1.00**	0.18	**1.02**	0.27	*0.99*
GM-CSF	**0.37**	0.20	**0.34**	0.06	**0.28**	0.04	*0.55*
RANTES	**0.29**	0.06	**0.40**	0.13	**0.40**	0.09	*0.22*
TIMP-1	**0.46**	0.28	**0.75**	0.43	**0.96**	0.94	*0.54*
TNF-*α*	**0.10**	0.03	**0.12**	0.03	**0.13**	0.04	*0.38*
VEGF	**0.56**	0.17	**0.41**	0.02	**0.49**	0.03	*0.07*
CXCl5	**0.71**	0.1	**1.1**	0.31	**0.72**	0.15	*0.07*
MIG	**0.36**	0.07	**0.35**	0.04	**0.33**	0.07	*0.81*
IL-13	**0.26**	0.17	**0.18**	0.04	**0.22**	0.05	*0.57*
IL-17	**0.22**	0.04	**0.24**	0.06	**0.31**	0.05	*0.10*
MIP-1*α*	**0.13**	0.02	**0.14**	0.01	**0.13**	0.03	*0.38*
MIP3*α*	**0.21**	0.04	**0.21**	0.01	**0.19**	0.02	*0.32*
IP-10	**0.27**	0.04	**0.33**	0.01	**0.30**	0.03	*0.06*
L-Selectin	**0.99**	0.18	**1.53**	0.32	**0.87**	0.20	0.01^*∗*^

IL-1*α*: interleukin-1 alpha; IL-1*β*: interleukin-1 beta; IL-1ra: interleukin-l receptor antagonist; IL-2: interleukin-2; IL-3: interleukin-3; IL-4: interleukin-4; IL-6: interleukin-6; IL-10: interleukin-10; CNTF: ciliary neurotrophic factor; sICAM: soluble intercellular adhesion molecule; CINC-1: Cytokine-Induced Neutrophil Chemoattractant-1; CINC-2: Cytokine-Induced Neutrophil Chemoattractant-2; CINC-3: Cytokine-Induced Neutrophil Chemoattractant-3; IFN-*γ*: interferon-gamma; GM-CSF: granulocyte macrophage colony stimulating factor; RANTES: regulated upon activation, normal T-cell expressed and presumably secreted; TIMP-1: tissue inhibitor of metalloproteinases-1; TNF-*α*: tumour necrosis factor; VEGF: vascular endothelial growth factor; CXCl5: lipopolysaccharide-induced CXC; MIG: monokine induced by gamma interferon; IL-13: interleukin-13; IL-17: interleukin-17; MIP-1*α*: Macrophage Inflammatory Protein-1 alpha; MIP-3*α*: Macrophage Inflammatory Protein-3 alpha; IP-10, interferon-gamma-induced protein-10 kDa/CXC motif chemokine 10.

## Abbreviations

CNS: Central nervous system
DPN: Distal sensory polyneuropathy
GABA: *γ*-Aminobutyric acid
IBA1: Ionized calcium binding adaptor molecule 1
PBS: Phosphate buffered saline
STZ: Streptozotocin.
